# Pediatric oral antihypertensive agents: analysis of prescription patterns and patient characteristics in a real-world study

**DOI:** 10.3389/fped.2026.1764582

**Published:** 2026-03-13

**Authors:** Haixin Cheng, Ying Cui, Ziyin Ma, Siyuan Wang, Xuli Zhong, Jianmin Zhang

**Affiliations:** 1 Department of Pharmacy, Capital Center for Children's Health, Capital Medical University, Capital Institute of Pediatrics, Beijing, China; 2 Department of Science and Technology, Capital Medical University, Beijing, China; 3 B.E. Candidate in Department of Bioengineering, University of Washington, Seattle, WA, United States

**Keywords:** antihypertensive medicines, children, drug utilization, hypertension, prescription

## Abstract

**Objectives:**

The aim of this study was to assess prescribing intensity and rational dosing of antihypertensives in children using Defined Daily Dose (DDD) and Drug Utilization Index (DUI).

**Methods:**

A retrospective cross-sectional analysis was conducted on all antihypertensive prescriptions dispensed at a tertiary children's hospital from May 2023 to April 2024, excluding those with incomplete data or on fixed-dose combinations. Prescription medicine use is basically reasonable when DUI is close to 1 (0.9–1.1), a DUI >1.1 suggests that the actual daily dose exceeds DDD, while a DUI <0.9 indicates underdosing. Statistical analysis was performed using SPSS 23.0 with significance at *p* < 0.05.

**Results:**

A total of 1,562 prescriptions for 422 children (12–18 years; 76.30% male) were analysed; prevalence peaked at 13 years. Among over-12-year-olds, DUI <0.9 for β-blockers, spironolactone and nifedipine; DUI ≈ 1 for furosemide, captopril and losartan; DUI >1.1 for hydrochlorothiazide, amlodipine, ramipril and fosinopril. Amlodipine comprised 42.8% of total DDDs, followed by fosinopril (25.6%) and ramipril (20.0%). Males showed borderline higher DUI values.

**Conclusions:**

Hypertension was most prevalent among 13- to 14-year-old, who also exhibited the highest antihypertensive exposure and drug-use intensity relative to girls. Once-daily formulations accounted for the majority of prescriptions. Pediatric oral hypertension dosing remains inconsistent in clinical practice. These findings support sex-specific management and guideline updates to improve blood pressure control in high-risk adolescents.

## 1 Introduction

Hypertension in children is becoming an increasingly serious public health issue in China. The prevalence of hypertension is 4% in the 6–17 years (5% in boys, and 3% in girls) (1). An increase in body fat is the primary contributing factor to hypertension (2). Non-pharmacological interventions, such as lifestyle modifications, are typically recommended as first-line treatment for pediatric hypertension. However, a significant proportion of children will eventually require drug therapy to effectively manage their BP, especially those with evidence of target organ damage at presentation (3).

Even though the use of antihypertensive medicines in children has increased significantly over the past several years, individualized dose calculations based on weight, age, or body surface area remain limited. For instance, Angiotensin-Converting Enzyme Inhibitors (ACE inhibitors) and Angiotensin II Receptor Blockers (ARBs) are among the most commonly prescribed antihypertensive agents for pediatric patients. In the 2017 American College of Cardiology (ACC)/American Heart Association (AHA) (4) and the 2022 European Society of Cardiology (ESC) guidelines on hypertension in children (5), ACE inhibitors and ARBs are both strongly recommend as first-line agents for Initial hypertension treatment. However, dosages for children are rarely approved by the U.S. Food and Drug Administration (FDA) or included in drug labeling (6). The 2016 European Society of Hypertension (ESH) guidelines provide explicit recommendations for starting and maximum doses based on body weight, aiming to promote standardized and individualized treatment (7). However, off-label drug use remains inevitable in practice, which may lead to a series of drug safety problems. The most commonly reported medication errors in pediatrics involve dosing and often arise from miscalculations of doses and dosing intervals (8), highlighting a considerable discrepancy between the treatment strategies recommended by authoritative guidelines and routine clinical practice.

The index of drug intensity based on DDD is proposed by the World Health Organization (WHO). However, the application of DDD in pediatric populations is limited due to the unique physiological and pharmacokinetic characteristics of children. This study aims to scientifically and accurately analyze the current situation of antihypertensive medicine use in children, to establish a methodology for evaluating these medicines in children, and to provide a reference for rational clinical drug use.

## 2 Methods

### 2.1 Study design and population

The data for this study were obtained from a hospital-based cross-sectional survey. The Children's Hospital of the Capital Institute of Pediatrics, a pediatric teaching hospital, was selected as the sampling site using a random sampling method. All prescriptions for antihypertensive therapy to children hospitalized between May 2023 and April 2024 were collected via the hospital information system. This is a platform with preset safeguards to protect the patients' private information. The study was approved by the Medical Ethics Committee of Children's Hospital, Capital Institute of Pediatrics (Approval Number: SHERLL2024077) according to *Measures for Ethical Review of Life Sciences and Medical Research Involving Humans in China*, and all patient privacy was strictly protected throughout the research process.

All relevant prescriptions that included a Western medicine diagnosis of hypertension were eligible for inclusion. The patients were children (12–18 years old) who met the diagnostic criteria for hypertension as defined by the 2017 American Academy of Pediatrics Guidelines for the Management of Hypertension (i.e., systolic and/or diastolic blood pressure ≥the 95th percentile for age, sex, and height on at least three separate occasions) (4). Drug categories were classified according to the Anatomical, Therapeutic, and Chemical (ATC) classification system. Prescriptions with missing information, abnormal dosage (according to the Drug Package Inserts or clinical guidelines), administration frequencies marked as “ONCE”, “pro re nata (PRN, as needed)”, or “immediately (ST)”, as well as fixed-dose combination products were excluded from the analysis (9).

### 2.2 Drug use research

The DDD method was used to analyze the rationality of drug dosages. DDD is defined as the average maintenance dose per day for a drug when used for its main indication in adults. DDDs provide a fixed unit of measurement independent of price, currency, package size and drug strength. These enable researchers to assess trends in drug consumption and to perform comparisons across different population groups (10).

The drug utilization index (DUI) was employed as a quantitative indicator to assess the rationality of medication use (9). Prescription medicine use is basically reasonable when DUI is close to 1. Considering the influence of other factors during the medication administration, this study regarded DUI as close to 1 when it ranged between 0.9 and 1.1. A DUI >1.1 suggests that the actual daily dose of a given drug exceeds DDD and potentially indicating drug overuse. Conversely, A DUI <0.9 indicates that the actual daily dose of this drug is lower than DDD, which may compromise treatment efficacy due to insufficient dosing (11). DDDs = drug consumption (mg)/DDD; DUI = DDDs/actual medication days.

Pediatric patients were the main subjects of this study. Although their doses should be calculated based on weight, body surface area, or age (12), amlodipine, nifedipine, metoprolol, propranolol, fosinopril, captopril, and spironolactone may reach adult doses by the age of 12 (13). Hence, the WHO DDD of antihypertensive drugs can be used in this drug utilization study among individuals aged 12 years and above.

### 2.3 Rationality evaluation

The recommended maintenance dose (long-term therapeutic dose) is usually preferred when establishing the DDD (10). To avoid underestimating consumption, fixed-dose combinations and prescriptions labeled “ONCE,” “PRN,” or “ST” were excluded.

### 2.4 Statistical analysis

The prescription data were arranged and processed using Microsoft Excel 2019 software. Factors related to patient age, gender, drug class, specification and actual medication days were included, and the baseline data table of patients was drawn for the corresponding calculation. Statistical analysis was conducted in SPSS 23.0 software. Differences were considered clinically significant when *p* < 0.05.

## 3 Results

### 3.1 Patient characteristics

A total of 1,102 prescriptions for children aged 12 years and older were extracted as the final data set. After data collation to obtain patient baseline information, the distribution of pediatric hypertension cases by age group and etiology was analyzed (Table 1). Among the 422 hypertensive children (age range: 12.0–17.9 years), there were 322 boys and 100 girls, yielding in a male-to-female ratio of 3:1 (76.30% vs. 23.70%). With the growth of age, the number of children with hypertension gradually increased, peaking at 13–14 years (*n* = 109, 25.83%) before gradually declining.

**Table 1:** Distribution of pediatric hypertension cases by Age group and etiology.

Age (years)	Total hypertension cases (n)	Boy *n* (%)		Girl *n* (%)		Total (%)	Primary hypertension *n* (%)		Secondary hypertension (%)		Primary comorbidities
12–13	78	57	13.51%	21	4.98%	18.48%	57	73.08%	21	26.92%	IgA Nephropathy, Nephrotic Syndrome, Lupus Nephritis, Henoch-Schönlein Purpura Nephritis
13–14	109	79	18.72%	30	7.11%	25.83%	94	86.24%	15	13.76%	IgA Nephropathy, Henoch-Schönlein Purpura Nephritis
14–15	91	76	18.01%	15	3.55%	21.56%	76	83.52%	15	16.48%	IgA Nephropathy, Henoch-Schönlein Purpura Nephritis
15–16	76	62	14.69%	14	3.32%	18.01%	72	94.74%	4	5.26%	IgA Nephropathy, Lupus Nephritis
16–17	44	30	7.11%	14	3.32%	10.43%	38	86.36%	6	13.64%	Henoch-Schönlein Purpura Nephritis
17–18	24	18	4.27%	6	1.42%	5.69%	22	91.67%	2	8.33%	IgA Nephropathy, Nephrotic Syndrome
Total	419	322	76.30%	100	23.70%	100.00%	356	84.96%	63	15.04%	

### 3.2 Research on drug utilization trend

Prescriptions for children aged over 12 years old involved 5 categories and 11 commonly used oral antihypertensive medications. The kinds of oral antihypertensive prescribed, DDD of antihypertensive medicines, the age of patients, drug consumption, medication time, the corresponding DDDs and DUI are presented in Table 2.

**Table 2:** Drug utilization analysis in children over 12 years old.

Drug	DDD (mg)	Age (years)	drug consumption (mg)	Medication time (day)	DDDs	Proportion (%)	DUI
Diuretics							
Hydrochlorothiazide	25		2,500	33.33	100.00	0.23%	3.00
		12	2,500	33.33	100.00	0.23%	3.00
Furosemide	40		2,000	50.00	50.00	0.12%	1.00
		12	2,000	50.00	50.00	0.12%	1.00
Spironolactone	75		14,000	316.67	186.67	0.44%	0.59
		12	8,000	166.67	106.67	0.25%	0.64
		13	2,000	50.00	26.67	0.06%	0.53
		14	4,000	100.00	53.33	0.13%	0.53
Beta blocking agents							
Metoprolol	50		83,000	2,400.24	1,660.00	3.90%	0.69
		12	13,500	410.24	270.00	0.63%	0.66
		13	16,000	390.00	320.00	0.75%	0.82
		14	30,500	876.67	610.00	1.43%	0.70
		15	9,500	313.33	190.00	0.45%	0.61
		16	10,500	340.00	210.00	0.49%	0.62
		17	3,000	70.00	60.00	0.14%	0.86
Propranolol	160		5,000	216.67	31.25	0.07%	0.14
		15	2,000	83.33	12.50	0.03%	0.15
		16	3,000	133.33	18.75	0.04%	0.14
Calcium channel blockers							
Amlodipine	5		91,105	13,255.67	18,221.00	42.76%	1.37
		12	13,440	2,023.00	3,192.00	7.49%	1.58
		13	27,090	4,027.33	5,425.00	12.73%	1.35
		14	23,765	3,272.50	4,557.00	10.69%	1.39
		15	14,910	2,262.17	2,863.00	6.72%	1.27
		16	7,735	1,060.50	1,323.00	3.10%	1.25
		17	4,165	610.17	861.00	2.02%	1.41
Nifedipine	30		33,500	4,708.33	1,116.67	2.62%	0.24
		12	9,500	1,566.67	316.67	0.74%	0.20
		13	8,500	1,150.00	283.33	0.66%	0.25
		14	5,500	641.67	183.33	0.43%	0.29
		15	5,000	633.33	166.67	0.39%	0.26
		16	3,000	366.67	100.00	0.23%	0.27
		17	2,000	350.00	66.67	0.16%	0.19
ACE inhibitors							
Ramipril	2.5		21,280	3,666.40	8,512.00	19.98%	2.32
		12	6,020	929.37	2,408.00	5.65%	2.59
		13	5,390	996.37	2,156.00	5.06%	2.16
		14	5,985	942.67	2,394.00	5.62%	2.54
		15	2,905	471.33	1,162.00	2.73%	2.47
		16	735	308.00	294.00	0.69%	0.95
		17	245	44.33	98.00	0.23%	2.21
Fosinopril	15		1,63,520	7,364.00	10,901.33	25.58%	1.48
		12	7,420	441.00	494.67	1.16%	1.12
		13	18,620	1,114.17	1,241.33	2.91%	1.11
		14	38,360	1,664.83	2,557.33	6.00%	1.54
		15	46,620	1,824.67	3,108.00	7.29%	1.70
		16	35,840	1,464.17	2,389.33	5.61%	1.63
		17	16,660	855.17	1,110.67	2.61%	1.30
Captopril	50		25,000	544.44	500.00	1.17%	0.92
		12	2,500	50.00	50.00	0.12%	1.00
		13	5,000	150.00	100.00	0.23%	0.67
		14	15,000	244.44	300.00	0.70%	1.23
		16	2,500	100.00	50.00	0.12%	0.50
ARBs							
Losartan	50		66,500	1,181.67	1,330.00	3.12%	1.13
		12	9,450	203.00	189.00	0.44%	0.93
		13	14,700	277.67	294.00	0.69%	1.06
		14	24,500	423.33	490.00	1.15%	1.16
		16	9,100	149.33	182.00	0.43%	1.22
		17	8,750	128.33	175.00	0.41%	1.36

The total antihypertensive medicines use expressed in DDDs among the medicine varieties differed significantly, ranging from 31.25 DDDs in propranolol to 18,221.00 DDDs in amlodipine. We also noted significant differences in DDDs across age groups for the same agents. For example, the DDDs of amlodipine varied by a factor of 6.3 between 13-year-olds (highest use: 5,425.00 DDDs) and 17-year-olds (lowest use: 861.00 DDDs).

**Figure 1:**
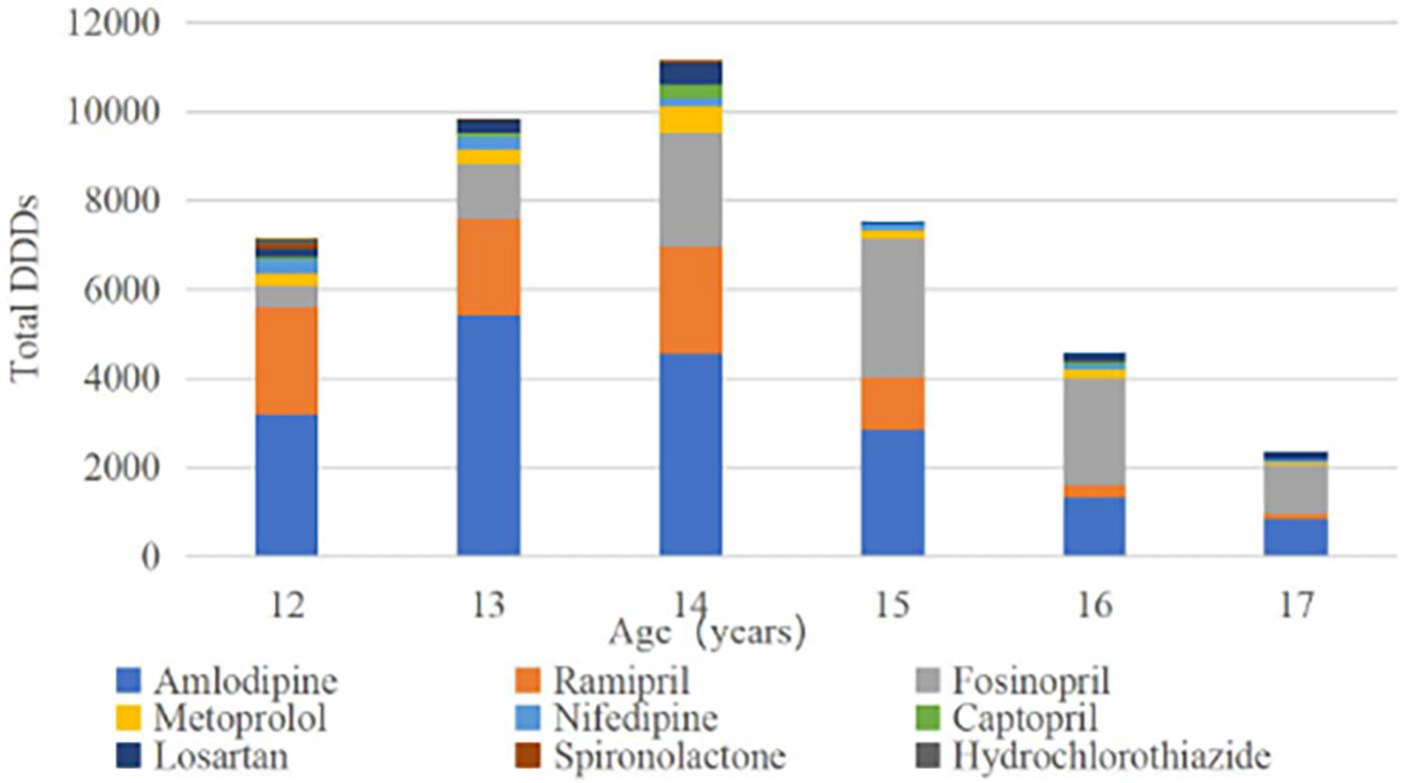
Total DDDs of antihypertensive medicine use in children 12–17 years of age.

Figure 1 shows the fluctuation in total outpatient antihypertensive DDDs use among individuals aged 12 years and older. Total DDDs were lowest in 17-year-olds, moderate in 12-, 15-, and 16-year-olds, and highest in 13- to 14-year-olds. Amlodipine, fosinopril, and ramipril were the most commonly used antihypertensives in all age groups. The highest proportion of total DDDs was attributed to amlodipine (42.76%), followed by fosinopril (25.58%), and ramipril (19.98%).

### 3.3 Drug intensity analysis

The DUI of β-blockers (metoprolol = 0.69; propranolol = 0.14), spironolactone (0.59), and nifedipine (0.24) was much less than 1. The index of furosemide (1.00), captopril (0.92), and losartan (1.13) was close to 1. The index of hydrochlorothiazide (3.00), amlodipine (1.37), ramipril (2.32), and fosinopril (1.48) was basically greater than 1.

For the most commonly used antihypertensive medicines—amlodipine, fosinopril, and ramipril—we statistically analyzed DUI to determine the relationship between patients' gender and the drug use intensity (Supplementary Table S1).

**Figure 2:**
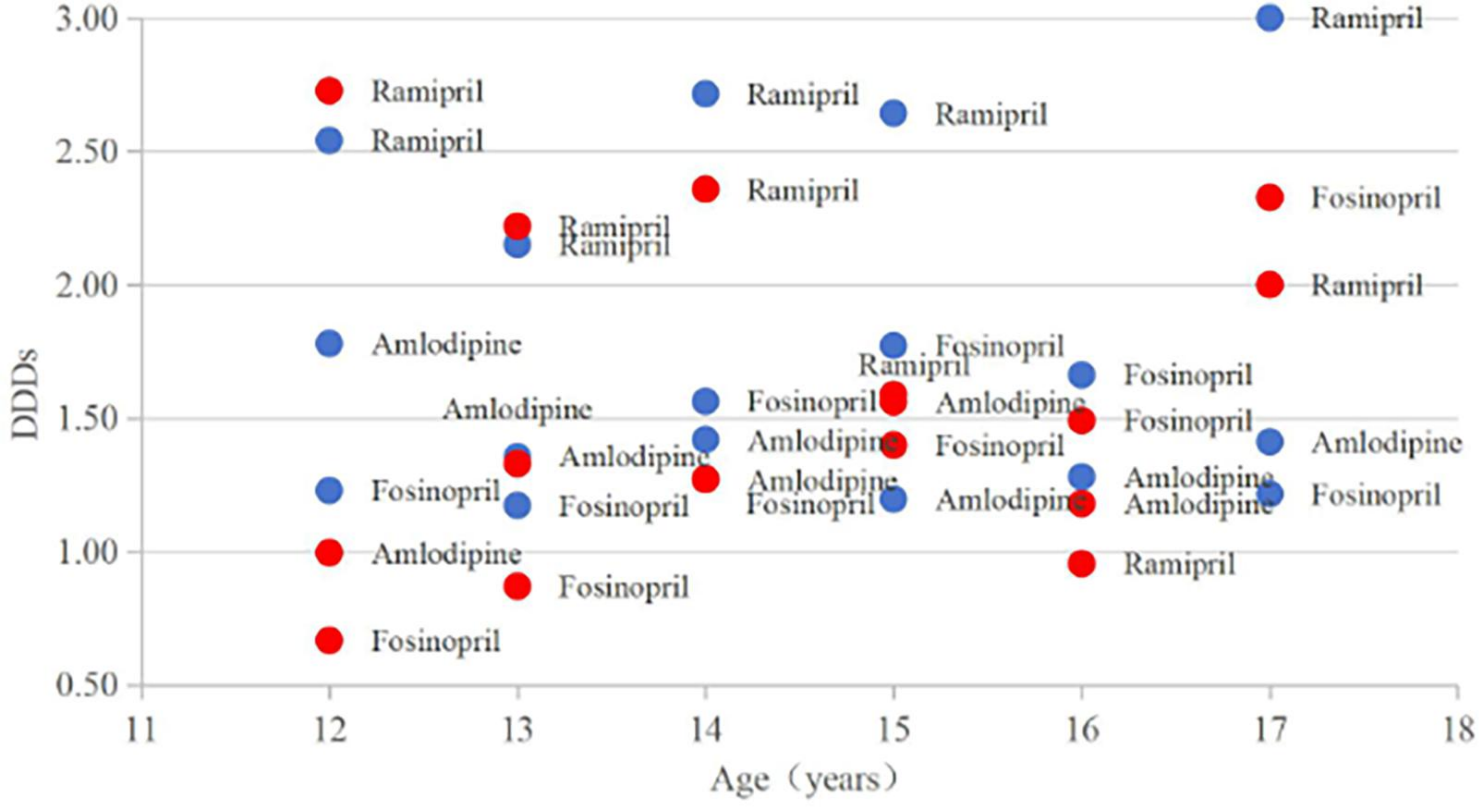
Correlation between DUI and sex in patients taking amlodipine, ramipril, or fosinopril girls are labellwd in red, boys in blue. DUI, the durg utilization index.

Our comparative analysis of the boy group (*n* = 16) and the girl (*n* = 16) group demonstrated marginally significant difference (independent *t*-test: *t*_30_ = 1.82, *p* = 0.078; Cohen's *d* = 0.64, medium effect size), with the boy group showing higher values (1.80 ± 0.66 vs. 1.54 ± 0.63). Parametric assumptions were validated through Shapiro–Wilk tests (males: *W* = 0.93, *p* = 0.22; females: *W* = 0.90, *p* = 0.08) and Levene's test confirmed variance homogeneity (*F*_1_, *t*_30_ = 0.41, *p* = 0.53). The non-parametric Mann–Whitney *U* test corroborated these findings (*U* = 98.0, *p* = 0.085), supporting the robustness of our results despite borderline significance. Notably, the highest DUI values (>2.50) were primarily observed in boys [*n*(boys) = 4; *n*(girls) = 1], while the lowest DUI values (<1.00) were more common in girls [*n*(boys) = 0; *n*(girls) = 4], in the range of 1–2.5, the proportion of boys and girls was equivalent [*n*(boys) = 12; *n*(girls) = 11] (Figure 2). The application analysis data of amlodipine, fosinopril and ramipril by age and gender are shown in Supplementary Table S2.

## 4 Discussion

### 4.1 Patient population analysis

This study reports the annual consumption and consumption intensity of antihypertensive medicines across pediatric age and gender groups in a children's hospital setting.

Analysis of the patient population based on prescription data revealed that boys have a higher incidence than girls. The incidence is associated with gender, as previously reported in descriptive studies (1, 4). In this study, to our knowledge, we show for the first time that adolescents, particularly those aged 13–14 years, account for the highest prevalence of hypertensive cases (25.83%), with significantly higher medicine consumption compared to other age groups.

The peak in hypertension prevalence may be partially attributed to the concurrent rise in both primary and secondary hypertension diagnoses during this period. This likely reflects the complex interplay of physiological changes during adolescence, which may exacerbate underlying susceptibility to both hypertension subtypes. However, given the cross-sectional nature of our data, causal relationships cannot be established, and further longitudinal studies are warranted to investigate this observation. Additionally, this peak likely reflects intensified clinical screening (e.g., school physical examination) in early adolescence, differing from epidemiological studies measuring total prevalence. The subsequent decline in prevalence among older adolescents may be explained by two interrelated factors: (a) a more active lifestyle and nonpharmacologic interventions in older adolescents, and (b) the potential discontinuation of treatment in some patients with isolated systolic hypertension in the young (ISHY) as they transition to adulthood. This explanation is consistent with some evidence indicating that ISHY may be physiological rather than pathological and usually resolves by adulthood (14). However, implementing pharmacologic intervention earlier and performing regular BP screening can also reduce pediatric target-organ damage (15).

### 4.2 Drug consumption analysis in different categories

Antihypertensive medication consumption is not uniform across different categories. Once-daily administration of medicines such as amlodipine, ramipril, and fosinopril shows the highest consumption rates (amlodipine: 42.76%; fosinopril: 25.58%; ramipril: 19.98%).

Once-daily administration of medicines improves adherence. If more than one medicine is required, they should be administered as separate formulations to facilitate individual dose titration, but fixed-dose combination products may be beneficial for adolescents if compliance is a problem (16).

### 4.3 Drug utilization analysis in different categories

In the present study, we introduced an age limitation of 12 years and older to analyze medication use status amongst children by dividing them into different age and sex groups. The DDD of a medicine is a useful tool for measuring the intensity of medication a person is taking, even when patients receive different classes of antihypertensives at varying doses. By expressing drug intensity numerically, we can conjecture the difficulty of BP control with pharmacotherapy.

These findings suggest that clinicians prescribed β-blockers, spironolactone, and nifedipine at conservative dosages. The use of furosemide, captopril, and losartan was appropriate, while the use of hydrochlorothiazide, amlodipine, ramipril, and fosinopril were prescribed at higher-than-recommended doses.

Physicians' prescribing patterns vary with characteristics of pharmacological properties of therapeutic agents. In general, the dose of β-blocker does not have to be high to achieve the therapeutic efficacy. This observation might be due to their potential adverse effects, such as the blockage of beta-adrenoceptors in the heart, peripheral vasculature, and liver. Additionally, it could also be attributed to the common practice of combining β-blockers with other antihypertensive agents in low-dose regimens, which aims to optimize therapeutic outcomes while minimizing side effects.

Medications fluctuated reasonably in CCBs. The short-acting oral formulation nifedipine was commenced on the temporary lowest recommended dose for rapid BP control. Following stabilization and identification the appropriate agent, the child should be prescribed amlodipine, a long-acting dihydropyridine CCBs, as the maintenance therapy. The dosage of amlodipine should be gradually increased (typically 2.5 mg every 2–4 weeks) until the BP falls within the target range or the maximum recommended dose (10 mg daily in pediatric patients) is attained.

In ACE inhibitors and ARBs, an overdose phenomenon was observed. Despite lacking formal approval from both FDA and the Chinese health authorities for pediatric patients with hypertension, ramipril is widely used off-label in children over 12 years old in clinical practice. The dosage of ramipril in pediatric patients is typically referenced against the adult dose, which often substantially far beyond DDD. Diuretic selection should be targeted to the child's underlying pathophysiology and the presence of concurrent disorders, resulting in wide variations in DUI.

### 4.4 Drug utilization analysis in different genders

We found an association between changes in DUI and sex. Small increases were observed in boys, and these results were of borderline significance. Sex differences in BP among children and adolescents are well known, with girls generally exhibiting lower BP levels compared to boys (17). During adolescence, these sex-based differences become more pronounced, a phenomenon closely linked to variations in body composition and the relationships of individual body compartments to BP (18). The innovative application of the DUI metric in this study further reveals that boys exhibit higher drug use intensity than girls. Consequently, it can be inferred that, in the context of drug use, boys may face greater challenges in BP control compared to girls.

Our study has limitations. DDD does not represent clinical use exactly, but represents one proxy. A descriptive overview study focusing only on antihypertensive prescribing in pediatric practice is subject to the inherent limitations of such an analysis. The analysis examined antihypertensive medication patterns in children aged 12 years and older; however, it did not adjust for potential confounding factors such as body weight, hypertension etiology, or concurrent therapies, all of which may influence treatment outcomes.

## 5 Conclusions

Our study demonstrates that 13- to 14-year-old boys had the highest prevalence of hypertension and the greatest antihypertensive use; blood pressure control appears to be more challenging in this group. Therefore, sex-stratified treatment protocols warrant investigation to improve blood pressure control in adolescent males. Once-daily antihypertensives dominated pediatric use.

Our data also indicate a gap between evidence and practice in pediatric antihypertensive prescribing. Based on this evidence, it may be valuable to consider an adjustment of the guidelines that both encourage the use of underutilized first-line therapies (e.g., thiazides) and, for agents with proven real-world efficacy (e.g., ramipril), formally recommend dosing regimens and emphasize adherence to the recommended maximum dose. This approach is designed to ensure optimal therapeutic outcomes while controlling adverse reactions.

## Data Availability

The raw data supporting the conclusions of this article will be made available by the authors, without undue reservation.
