# Supplementary material for: Pediatric oral antihypertensive agents: analysis of prescription patterns and patient characteristics in a real-world study
Source: Front Pediatr. 2026 Mar 13;14:1764582. doi: 10.3389/fped.2026.1764582 (PMC13021652; doi:10.3389/fped.2026.1764582)
Supplement: Supplementary file 1 [file Supplementaryfile1.docx]

**Supplementary Materials:** TABLE S1: Gender-based comparison of DUI (amlodipine, ramipril, and fosinopril), TABLE S2: Application analysis of amlodipine, fosinopril and ramipril by age and gender.

**TABLE S1** | **Gender-based comparison of DUI (amlodipine, ramipril, and fosinopril)**

| **Test Category** | **Parameter** | **Boy Group** | **Girl Group** | **Statistical Results** |
| --- | --- | --- | --- | --- |
| **Descriptive Statistics** | Mean ± SD | 1.80 ± 0.66 | 1.54 ± 0.63 | - |
| **Normality Test** | W statistic | 0.931 | 0.901 | - |
| (Shapiro-Wilk) | p-value | 0.221 | 0.078 |  |
| **Homogeneity of Variance** | F statistic | - | - | F (1,30) = 0.412 |
| (Levene's Test) | p-value |  |  | p = 0.526 |
| **Primary Analysis** | t statistic | - | - | t (30) = 1.823 |
| (Independent t-test) | df |  |  | d = 0.644 |
|  | p-value |  |  | p = 0.078* |
| **Robustness Check** | U statistic | - | - | U = 98.0 |
| (Mann-Whitney U) | p-value |  |  | p = 0.085 |

*p < 0.1, **p < 0.05

SD = standard deviation

**TABLE** **S2** | **Application analysis of amlodipine, fosinopril and ramipril by age and gender**

| **Drug** | **Age**  **(years)** | **DDDs Boy** | **DDDs Girl** | **Medication time**  **(day) Boy** | **Medication time**  **(day) Girl** | **DUI Boy** | **DUI Girl** |
| --- | --- | --- | --- | --- | --- | --- | --- |
| **Amlodipine** |  |  |  |  |  |  |  |
|  | 12 | 2674.00 | 518.00 | 1502.67 | 520.33 | 1.78 | 1.00 |
|  | 13 | 3654.00 | 1771.00 | 2695.00 | 1332.33 | 1.36 | 1.33 |
|  | 14 | 3815.00 | 742.00 | 2688.00 | 584.50 | 1.42 | 1.27 |
|  | 15 | 2191.00 | 672.00 | 1831.67 | 430.50 | 1.20 | 1.56 |
|  | 16 | 917.00 | 406.00 | 716.33 | 344.17 | 1.28 | 1.18 |
|  | 17 | 861.00 |  | 610.17 |  | (1.41) |  |
| **Ramipril** |  |  |  |  |  |  |  |
|  | 12 | 1722.00 | 686.00 | 677.83 | 251.53 | 2.54 | 2.73 |
|  | 13 | 1722.00 | 434.00 | 800.83 | 195.53 | 2.15 | 2.22 |
|  | 14 | 1302.00 | 1092.00 | 479.50 | 463.17 | 2.72 | 2.36 |
|  | 15 | 1036.00 | 126.00 | 392.00 | 79.33 | 2.64 | 1.59 |
|  | 16 |  | 294.00 |  | 308.00 |  | (0.95) |
|  | 17 | 28.00 | 70.00 | 9.33 | 35.00 | 3.00 | 2.00 |
| **Fosinopril** |  |  |  |  |  |  |  |
|  | 12 | 438.67 | 56.00 | 357.00 | 84.00 | 1.23 | 0.67 |
|  | 13 | 1054.67 | 186.67 | 899.50 | 214.67 | 1.17 | 0.87 |
|  | 14 | 2361.33 | 196.00 | 1510.83 | 154.00 | 1.56 | 1.27 |
|  | 15 | 2641.33 | 466.67 | 1491.00 | 333.67 | 1.77 | 1.40 |
|  | 16 | 2006.67 | 382.67 | 1207.50 | 256.67 | 1.66 | 1.49 |
|  | 17 | 961.33 | 149.33 | 791.00 | 64.17 | 1.22 | 2.33 |

Values in parentheses were not included in the calculation.
